# Influence of rewetting on microbial communities involved in nitrification and denitrification in a grassland soil after a prolonged drought period

**DOI:** 10.1038/s41598-018-38147-5

**Published:** 2019-02-19

**Authors:** Verena Hammerl, Eva-Maria Kastl, Michael Schloter, Susanne Kublik, Holger Schmidt, Gerhard Welzl, Anke Jentsch, Carl Beierkuhnlein, Silvia Gschwendtner

**Affiliations:** 1 Research Unit Comparative Microbiome Analysis - Helmholtz Zentrum München, Ingolstädter Landstr. 1, 85764 Neuherberg, Germany; 2 Chair for Soil Ecology - Technische Universität München, Ingolstädter Landstr. 1, 85764 Neuherberg, Germany; 3 Institute of Natural Sciences - Universität Koblenz Landau, Campus Koblenz, Universitätsstraße 1, 56070 Koblenz, Germany; 4 Disturbance Ecology - University of Bayreuth, Universitätsstr. 30, 95447 Bayreuth, Germany; 5 Chair of Biogeography - University of Bayreuth, Universitätsstr. 30, 95447 Bayreuth, Germany

## Abstract

The frequency of extreme drought and heavy rain events during the vegetation period will increase in Central Europe according to future climate change scenarios, which will affect the functioning of terrestrial ecosystems in multiple ways. In this study, we simulated an extreme drought event (40 days) at two different vegetation periods (spring and summer) to investigate season-related effects of drought and subsequent rewetting on nitrifiers and denitrifiers in a grassland soil. Abundance of the microbial groups of interest was assessed by quantification of functional genes (*amoA*, *nirS*/*nirK* and *nosZ*) via quantitative real-time PCR. Additionally, the diversity of ammonia-oxidizing archaea was determined based on fingerprinting of the archaeal *amoA* gene. Overall, the different time points of simulated drought and rewetting strongly influenced the obtained response pattern of microbial communities involved in N turnover as well as soil ammonium and nitrate dynamics. In spring, gene abundance of *nirS* was irreversible reduced after drought whereas *nirK* and *nosZ* remained unaffected. Furthermore, community composition of ammonia-oxidizing archaea was altered by subsequent rewetting although *amoA* gene abundance remained constant. In contrast, no drought/rewetting effects on functional gene abundance or diversity pattern of nitrifying archaea were observed in summer. Our results showed (I) high seasonal dependency of microbial community responses to extreme events, indicating a strong influence of plant-derived factors like vegetation stage and plant community composition and consequently close plant-microbe interactions and (II) remarkable resistance and/or resilience of functional microbial groups involved in nitrogen cycling to extreme weather events what might indicate that microbes in a silty soil are better adapted to stress situations as expected.

## Introduction

The IPCC report of 2014^1^ has indicated major changes related to climate in the northern hemisphere, especially in the precipitation variability, for the next 20 years. Major scenarios predict an increase in the frequency of long lasting drought periods, serious flooding or even both following each other^1–4^. These extreme fluctuations of water availability may lead to a reduction of the soil quality as well as the ecosystem benefits provided by soils which includes plant production or carbon sequestration. In this respect, the performance of microbes in soil and changes in both structure and function of the soil microbiome in response to shifts in the climatic conditions play a very important role as bacteria, fungi and archaea can be considered as the architects of the soil quality and catalyze most nutrient turnover processes^5^.

Several studies have shown that drought and decreased water potentials in soil act as severe stress factors for microbes^6–8^. The mobility of microbes as well as the substrate diffusion is reduced and therefore nutrient resources for microbes are limited under drought conditions^9^. As a consequence, most biogeochemical turnover processes are slowed down or stopped^7,8^. However, consequences of different drought time points during the vegetation period on the observed response pattern of the soil microbiome are not well understood. Waldrop and Firestone^10^ reported that the composition of microbial community shows seasonal changes and that these patterns are largely influenced by seasonal plant community effects and also by seasonal differences in soil moisture. Furthermore, Bardgett *et al*.^11^ mentioned an increasing amount of studies dealing with seasonal changes in root exudates and resulting correlations with belowground properties and plant nutrient supply, nutrient cycling and growth. These seasonal dynamics are important as they control the nutrient availability and interactions between plants and microbes.

We hypothesize that microbial response pattern seasonally differs to a large extent after rewetting due to seasonal changes in plant biomass, quality and amount of root exudates, turnover of organic matter and litter decomposition. In order to test this hypothesis, we performed a plot experiment in grassland soils with simulated extreme droughts followed by intensive rewetting. The drought was set either in May - June (D1) or July - August (D2). The microbial response pattern and the chemical soil properties were measured at the end of the drought period as well as one and two and four weeks after the rewetting event. For comparison, control plots were set up that did not undergo the drought/rewetting cycle. Ammonia oxidizing and denitrifying microbes were selected for the study in order to compare microbial groups with different physiologies. Ammonia oxidizers are autotrophs and catalyze the first step of nitrification (the oxidation of ammonia to nitrite, whereas denitrifiers are typical heterotrophic microbes which use nitrate or other forms of oxidized nitrogen as alternative electron acceptor in the absence of oxygen^12^. Both groups of microbes also differ in their diversity: ammonia oxidation can only be performed by a very small group of specialized bacteria and archaea (AOB and AOA) or by the recently discovered comammox bacteria belonging to the genus *Nitrospira*^13^, whereas in contrast many microbes in soil are capable for denitrification and phylogenetically widespread^14^. In this study, the abundance as well as the diversity of the microbial groups of interest were assessed using quantitative real-time PCR and terminal restriction fragment length polymorphism (t-RFLP) using marker genes typically for ammonia oxidizers or denitrifiers (*amoA*, *nirS*/*nirK* and *nosZ*).

## Results

### Soil water content

Before the simulation of the spring drought started in May, the soil moisture content of C and D1 plots was comparable (38 vol. %) which is equivalent to 95% of maximal water holding capacity (WHC_max_) (Fig. S1). During D1 the soil moisture constantly decreased to a minimum of 16 vol. % (40% WHC_max_), at the last day of the drought (Table 1), with a significant difference over time (p = 0.008), whereas in the control plots only a slight decrease due to the natural precipitation levels were observed. Within one week after rewetting, the soil moisture levels of D1 plots and C plots were comparable (27 vol. %; t1). A similar trend was observed during summer drought. At the end of June (before starting the drought simulation), the soil moisture of D2 and C plots was comparable (24 vol. %; 60% WHC_max_; Fig. S1). The simulated drought resulted in reduced soil moisture in D2 plots at the end of the drought period (13 vol. %; t0) and differed significantly (p = 0.002) from the control plots. The rewetting of D2 plots resulted in a significant increase (p = 0.000) of soil moisture levels (33 vol. %; 38% WHC_max_; t1) which was comparable to C plots (30 vol. %; t1).

**Table 1:** Soil moisture (SM) [vol%], Soil chemical properties [µg g^−1^ dw] and number of terminal restriction fragments (TRFs) of the archaeal *amoA* AOA gene for drought treatment and control in spring (D1 and C) and summer (D2 and C) over the sampling period.

	Time	Treatment	SM	NH_4_^+^-N	NO_3_^−^-N	TRFs
spring	29^th^ June	C	26.29 (12.01)^A^	2.05 (0.55)^A^	1.71 (1.47)^A^	52 (18)^A^
	(t0)	D1	16.02 (0.92)^a^	1.61 (0.23)^a^	0.74 (0.67)^a^	66 (8)^a^
	07^th^ July	C	28.79 (10.75)^A^	1.42 (0.26)^B^	2.88 (1.75)^A^	56 (10)^A^
	(t1)	D1	27.85 (7.78)^b^	1.12 (0.29)^b^	2.75 (1.30)^bc^	68 (15)^a^
	14^th^ July	C	28.87 (7.30)^A^	0.77 (0.02)^C^	2.52 (0.82)^A^	54 (6)^A^
	(t2)	D1	26.71 (7.83)^a^	0.76 (0.18)^b^	2.71 (0.53)^b^	59 (6)^a^
	28^th^ July	C	37.89 (5.42)^A^	1.42 (0.20)*^B^	3.79 (1.03)^A^	53 (20)^A^
	(t3)	D1	29.82 (8.00) ^b^	1.03 (0.25)*^b^	4.30 (0.80)^c^	54 (3)^a^
summer	10^th^ August	C	26.74 (3.48)*^AB^	0.98 (0.21)^A^	2.95 (1.34)^A^	51 (10)^A^
	(t0)	D2	13.24 (6.54)*^a^	0.81 (0.33)^a^	3.92 (1.99)^ab^	61 (13)^a^
	18^th^ August	C	29.77 (5.25)^B^	1.09 (0.24)^A^	3.67 (2.58)^A^	60 (8)^AB^
	(t1)	D2	33.17 (4.09)^b^	1.00 (0.31)^a^	2.80 (1.57)^a^	63 (10)^a^
	25^th^ August	C	21.31 (4.53)*^A^	1.03 (0.09)^A^	2.06 (0.90)*^A^	73 (14)^B^
	(t2)	D2	27.74 (2.13)*^b^	1.19 (0.16)^a^	4.09 (1.35)*^ab^	74 (10)^a^
	08^th^ September	C	24.82 (4.18)^AB^	1.06 (0.15)^A^	3.44 (2.24)^A^	63 (11)^AB^
	(t3)	D2	28.17 (3.77)^b^	0.94 (0.08)^a^	5.79 (1.33)^b^	54 (12)^a^

Standard deviations are shown in brackets. Significant differences (p < 0.05) between C and D1 or C and D2 are marked with asterisks and were calculated with a student’s t -test. Significant differences (p < 0.05) over time were calculated separately for C (capital letters), D1 and D2 (small letters) via repeated measurement ANOVA (Tukey-HSD) and are indicated by different letters.

### Soil chemical parameters

Highest ammonium concentrations were measured at t0 (end of the drought period; Table 1) in D1 (1.61 µg g^−1^ dw) and C plots (2.05 µg g^−1^ dw) plots and decreased significantly after rewetting. In contrast, nitrate concentrations increased significantly after rewetting up to 4.30 µg g^−1^ dw (t3) in D1 but remained constant at control plots (Table 1). For both ammonium and nitrate concentrations no treatment effect was observed when comparing D1 and C plots, except a temporary higher ammonium concentration in D1 at t3. In contrast to the spring drought, ammonium and nitrate concentrations during the summer drought were not affected by time or treatment (except a temporary higher nitrate concentration at D2 compared to C plots at t2), ranging from 0.81 µg g^−1^ dw (t0) to 1.19 µg g^−1^ dw (t2) for ammonium and 2.06 µg g^−1^ dw (t2) to 5.79 µg g^−1^ dw (t3) for nitrate.

### Abundance of ammonia-oxidizers and denitrifiers

**Figure 1:**
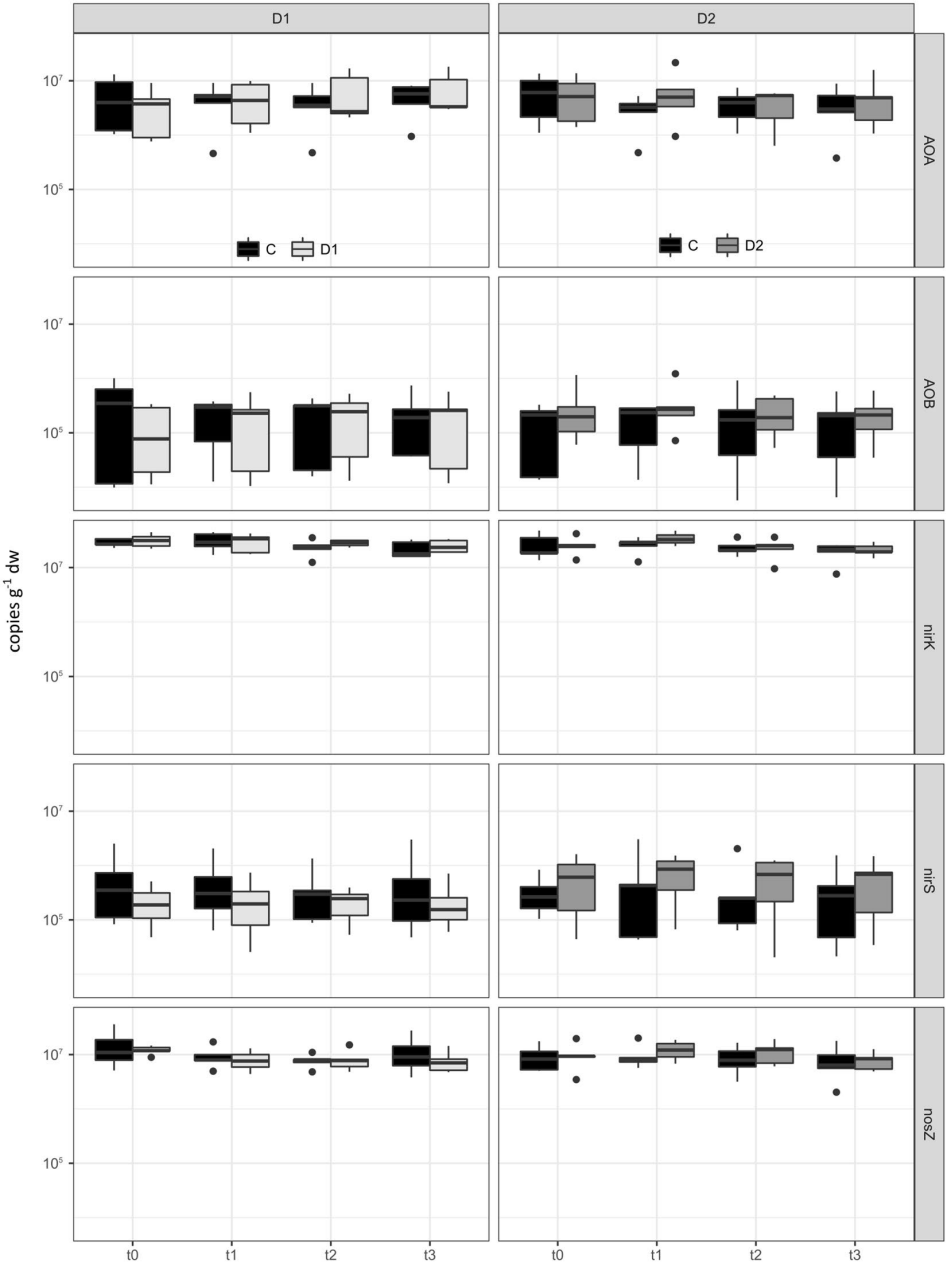
Gene abundance for archaeal and bacterial ammonia-oxidizers based on gene copy numbers of the *amoA* gene as well as for nitrite reducers harboring the *nirK* gene, nitrite reducers harboring the *nirS* gene and N_2_O reducers harboring the *nosZ* gene shown in gene copies g^−1^ dw. Left side shows spring drought event: bars for C (black) and D1 (light grey), right side shows summer drought event: bars for C (black) and D2 (dark grey). Sampling took place on the last day of the drought (t0), one (t1), two (t2) and four (t3) weeks after rewetting for D1 and D2, respectively.

The abundance of archaeal ammonia-oxidizers (AOA), remained constant during the sampling period for both control and drought/rewetting treatment in spring and summer (3.08–7.55 × 10^6^ copies g^−1^ dw; Fig. 1). Furthermore, the abundance of AOA was comparable between C and D plots. As expected, the number of bacterial ammonia-oxidizer (AOB) was approximately one order of magnitude lower compared to AOA and ranged between 1.47–4.04 × 10^5^ copies g^−1^ dw (Fig. 1). As for AOA, no significant differences between treatments for both, control and drought/rewetting in spring and summer were observed for AOB.

For denitrifiers, the abundance of *nirK* harboring nitrite reducers was about two orders of magnitude higher compared to the *nirS* harboring counterpart (1.98–3.47 × 10^7^ copies g^−1^ dw compared to 2.22–8.02 × 10^5^ copies g^−1^ dw; Fig. 1) during the whole experimental period independently from the two drought simulation treatments. Whereas, abundance of *nirK* harboring denitrifiers remained constant during time and did neither respond to D1 nor D2 treatment compared to the respective controls, *nirS* harboring nitrite reducers were irreversible reduced after spring drought (p > 0.05). Similar to the denitrifiers harboring the *nirK* gene, neither seasonal nor treatment related effects were observed for denitrifiers harboring the *nosZ* gene (6.65 × 10^6^–1.57 × 10^7^ copies g^−1^ dw). Overall the abundance of *nosZ* harboring nitrous oxide reducers was significantly lower than the abundance of nitrite reducers (*nirK* + *nirS*).

Overall, similar results were obtained when abundance data of ammonia oxidizers was related to ng of extracted DNA (data not shown).

### Diversity of ammonia-oxidizing archaea

**Figure 2:**
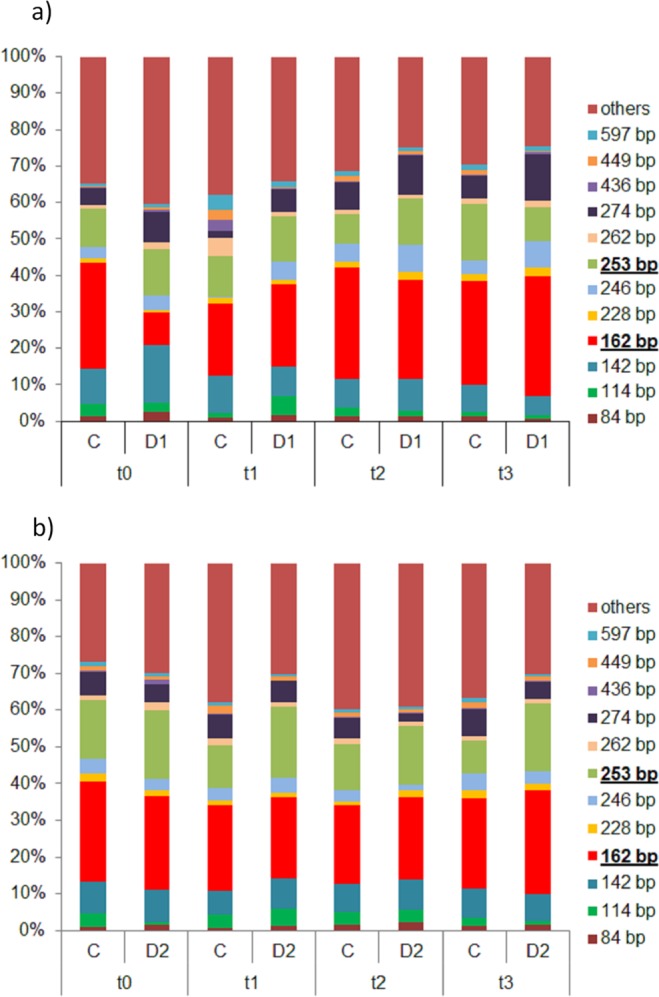
Relative abundance of the TRFs of the *amoA* AOA gene in percent [%] for (**a**) spring drought event (D1 and C) and (**b**) summer drought event (D2 and C). TRFs smaller than 3% are combined as “others”. Sampling took place on the last day of the drought (t0) and one (t1), two (t2) and four (t3) weeks after rewetting for D1 and D2, respectively. Most dominant peaks (162 and 253) are marked in the legend with bold and underlined letters.

The diversity analysis for the archaeal ammonia oxidizers based on the *amoA* gene revealed 104 different terminal restriction fragments (TRFs). Although not significant due to high replicate variability, a trend for higher TRF richness was observed for D1 (54–68 TRFs) compared to C (52–56 TRFs) plots (Table 1). AOA community profile in spring was dominated by four TRFs (142, 162, 253 and 274), accounting for 41–65% of total peak height (Fig. 2a). Three of them (TRF 142, 253 and 274) were positively affected by drought, showing a higher mean relative abundance at D1 compared to controls (p > 0.05). While TRFs 253 and 274 remained constant after rewetting, TRF 142 decreased significantly from 17 to 5% of total peak height at t3. In contrast, TRF 162 increased significantly after rewetting from 9 to 36% (t3). In silico analysis using amoA_AOA Feifei-Liu reference database of FunGene revealed that TRF 162 is most likely assigned to different members of Thaumarchaeota including *Candidatus* Nitrososphaera evergladensis, *Candidatus* Nitrosophaera viennensis, *Candidatus* Nitrosotalea devanaterra and *Candidatus*_Nitrosoarchaeum_limnia_SFB1) while TRF 253 might be assigned to *Nitrosopumilus maritimus* (group 1.1a) and *Candidatus* Nitrosotenuis. The same four fragments (142, 162, 253 and 274) dominated AOA terminal restriction length polymorphism (T-RFLP) profiles in summer, accounting for 52–67% of total peak height (Fig. 2b). In contrast to spring, no influence of drought and rewetting could be observed at D2.

## Discussion

### Effects of the drought simulations

Water contents in response to D1 and D2 treatments were highly reduced at the end of both drought periods, whereas, in the control plots the soil moisture contents were above 60% maximal water holding capacity (WHC_max_) at the same time points. Taking into account that a soil water content of around 60% WHC_max_ presents the best conditions for microbial activity in terms of oxygen- and water availability as well as nutrient distribution in upland soils^15^, the spring as well as the summer drought simulation in this study might have resulted in a potential improvement of the living conditions for oxidative and a potential worsening for reductive soil microbes. Considering that denitrification is a process to gain alternative electron acceptors under anoxic conditions, denitrifiers should be negatively affected by simulated drought events. Interestingly, we observed a reduced abundance of denitrifiers harboring the *nir*S gene but not of those caring the *nirK* and *nosZ* gene after spring drought (D1) compared to the control plots, suggesting a higher drought sensitivity for *nirS* harboring microorganisms. This is in accordance with Hartmann *et al*.^16^, showing that drought in pasture soils did not affect *nirK* and *nosZ* harboring microbes but decreased abundance of denitrifiers caring the *nirS* gene. Moreover, as this negative effect on *nirS* harboring denitrifiers was only found after spring but not after summer drought our results imply that these drought effects were strongly driven by the season. This might be explained by seasonal differences due to plant growth stage or changes in root exudates as mentioned earlier by Kaiser *et al*., Rasche *et al*. and Regan *et al*.^17–19^. During early summer times (end of D1 treatment) plants have a higher uptake rate of nitrogen to meet their growth demands and to build up biomass compared to late summer times and consequently compete highly with microbes for nitrogen^20,21^. Hence, less nutrients are available for microbes that lead, amongst others, to a decrease in gene abundances. These seasonal variations affect for example microbial community composition of denitrifying bacteria^22^ and may explain differences in the dynamics of *nirS* harboring denitrifiers after spring and after summer drought. Although *nosZ* abundance was not affected by drought it has to be considered that in the present study only the “classical” *nosZ* gene (*nosZ*I) was investigated. Recently, a new cluster of atypical *nosZ* genes, termed clade II *nosZ* genes, was discovered^23^ which encodes for enzymes catalyzing the same reaction but having <50% amino acid sequence similarity with type I NosZ enzymes. These *nosZ*II sequences are often at least as abundant as *nosZ*I in soil, suggesting that many N_2_O reducers were not covered in the present study. As clade II organisms were found to be more sensitive to environmental parameters^24^, potential drought effects might have been overlooked. However, *nosZ*II gene abundance is positively linked to pH^25^. Thus, this clade might be of minor importance in the studied ecosystem due to highly acidic soil (pH 4.1). Given the minor drought effect observed on denitrifier gene abundance and the results of previous studies showing that changing moisture regimes had only small or even no effect on denitrifier community^26,27^, denitrification was not investigated in more detail (e.g. by community fingerprinting) in the present study.

Contrary to denitrification, drought might be expected to increase oxidative processes like nitrification. Surprisingly, our results showed no change in abundance of AOA and AOB after spring and summer drought compared to the respective controls. This might be explained by the low soil pH of 4.1, which shifts the equilibrium between ammonia and ammonium towards ammonium^28^ (ratio of NH_3_/NH_4_^+^ = 1:20,000 at pH 5^29^ and p *K*_*a*_ for NH_4_^+^ ⇆ NH_3_ equilibrium is 9.25^30^), resulting in worse conditions for soil microbes preferring ammonia^31,32^. In addition, acid soils bind less positive loaded compounds like ammonium or ammonia^33^, what might be even more strengthened with drought. Further, low soil pH affects microbial activity more negative than plant activity^34^ and consequently favors plants acting as competitors for ammonium uptake with microbes^12,35,36^. This shift was found to be even more pronounced at simulated extreme drought events in grassland soil^6^. Although drought did not influence abundance of archaeal and bacterial ammonia oxidizers, this might not exclude shifts in diversity pattern. Coinciding with Gubry-Rangin *et al*.^37^, we observed that archaea dominated over bacteria in acid soil. Moreover, archaea were shown to be the main performer of nitrification in acidic soil^38^. Thus, we focused on community composition of AOA in our study. The predominant TRF 162 in all samples could be assigned (I) to a low pH adapted soil isolate *Candidatus* Nitrosotalea devanaterra, which has an growth optimum at pH 4.0–5.5 and is able to oxidize both ammonia and ammonium^29^ and (II) to different members of *Candidatus* Nitrososphaera, which are well adapted to environmental changes by formation of biofilms, detoxification and adhesion and thus dominates 15 out of 16 soils^39^. However, it has to be considered that assignment was based on in silico analysis using *amo*A_AOA reference database of FunGene without sequencing confirmation. Not only abundance but also community composition of ammonia oxidizing archaea remained unaffected by the drought simulations, which supports earlier observations on resistance of AOA towards drought stress^40^ and a good adaptation of AOA to a broad range of growing conditions and substrate concentrations^41,42^.

### Effects of rewetting

Rewetting was expected to improve the availability of soil water for microbial communities and consequently increase microbial growth. Several studies described an increase in C and N mineralization within one to four days after rewetting of dry soils^43–47^, which indicates a release of labile substrates due to microbial cell lysis or osmoregulation. This additional input of ammonium, nitrate or nitrite due to degraded biomass after rewetting might be expected to result in higher abundance of nitrifiers and denitrifiers. However, this could not be observed in the present study. Possible explanations for missing the momentary peak resulting from the fast turnover rate of N mineralization could be implicated (I) by our sampling time points, (II) the quantification of genes (DNA level) instead of transcripts (RNA level) which might have been correlated stronger to the real microbial activity and (III) by a strong competition between plants and microbes for ammonium and nitrate, supporting data from Bannert *et al*.^35^. Besides the potential positive effect of rewetting due to higher substrate input, oxidative processes like nitrification might be negatively influenced due to decreased oxygen diffusion. However, a decrease in archaeal and bacterial ammonia oxidizers with increasing soil moisture as described by Horz *et al*.^48^ was not observed in the present study, probably since the water content was not exceeding 70% WHC_max_ after rewetting. Furthermore, AOA can also act as heterotrophic microbe and hence not only use ammonia but also organic N as N source^31,49,50^. Interestingly, diversity pattern of AOA changed significantly after rewetting, although gene abundance remained constant. While the predominant TRF 162 increased, TRFs 142 and 84 decreased with increased soil moisture, indicating that some archaeal ammonia oxidizers might be better adapted to changing environmental conditions than others. Coinciding with responses to simulated drought events, the observed changes were much more pronounced after spring drought rewetting compared to rewetting after summer drought, which supports our hypothesis that microbial responses to extreme events are strongly dependent on the season due to plant derived effects as plant biomass, plant community composition and the physiological state of grassland on the experimental plots differ between the setting of the spring respectively summer drought. This seasonal impact was also reported by Grant *et al*.^51^ as changes in functional composition of the grassland could have been shown for spring but not for summer treatment within the present experiment. A significant increase (p < 0.001) in the aboveground net primary production (ANPP) of forbs and a significant decrease (p = 0.038) in ANPP of grasses gave evidence that the composition of species was significantly affected by the timepoint of the drought treatment. This was furthermore, confirmed by the functional group evenness that was significantly impacted by D1. A shift in the competitive balance from dominant grasses to subdominant forbs could be reported. As grasses have a shallow root system concentrated in the upper soil layer they seem to be more vulnerable to drought stress as forbs with a deeper root system. This change of root depth and therefore changes in root exudate and its composition can furthermore impact microbial communities.

## Conclusion

Our results indicate a strong seasonal dependency of microbial responses to extreme climatic events like drought and subsequent rewetting, which might be explained by plant derived effects, e.g. seasonally different plant development stage, plant community composition and, consequently, root exudate quantity and quality. However, further studies mainly simulating repeated drying-rewetting cycles are needed to draw conclusions for climatic modeling out of this dataset, as response pattern of microbes might differ after repeated stress simulation. Studies within a sandier soil texture, where water storage capacity is lower, should be done to compare and verify results related to weather extremes in different types of soil. In addition, this study was conducted with DNA, which serves as a proxy for the abundance of selected microbial groups; thus, further analysis with RNA can point out the expression of microbial genes that are involved in the metabolism of root exudates, soil processes and mineralization of nutrients. Moreover, additional genes should be analyzed to give a more detailed view of effects of changing soil water regimes on N cycling processes. Additionally, nitrification and denitrification assays should be performed to link genetic potential directly to enzyme activity. Overall, our two experiments indicate a high resilience and resistance to extreme weather events on the level of selected microbial groups linked to the nitrogen cycle that could be confirmed and can give evidence that microbes in a silty soil are better adapted to stress situations as expected.

## Methods

### Study site

The study was part of the EVENT 2 experiment at the Ecological-Botanical Garden of the University of Bayreuth, Germany (49°55′19″ N, 11°34′55″ E and 365 m above sea level)^52,53^. The plant community has been typical for semi-natural-grassland and was dominated by tall grasses, especially *Alopecurus pratensis* and *Arrhenatherum elatius*. The mean annual temperature was 8.2 °C and the mean annual precipitation of the region was 724 mm (1971–2000). The soil type has been classified as a Gleysol^54^. The experimental site was abandoned for 25 years before the experiment started without any plowing or addition of fertilizers. The A - horizon (0–30 cm depth) had a soil texture of 42% sand, 43% silt, 15% clay, a total carbon content of 2.34%, a total nitrogen content of 0.20%, a pH of 4.1 (1 M KCl), a water holding capacity of 40% and a permanent wilting point of 15%^55^.

### Experimental design and sampling

In this experiment we simulated the following treatments: spring drought (D1) (19^th^ May to 29^th^ June 2009), summer drought (D2) (30^th^ June to 10^th^ August 2009) and control (C). The experiments were set up using a latin square design, with a size of 1.5 × 1.5 m per plot^55^. Each treatment was replicated five times, resulting in 15 plots in total.

Each drought period lasted 42 days based on the duration of a statistically calculated local 1,000-years extreme event and was realized by using rain-out shelters as described by Walter *et al*.^53^. Briefly, rain-out shelters, starting in a height of 0.8 m above soil, were used to exclude rain but to enable ventilation and to avoid overheating of the shelters. The shelters were made of a transparent polyethylene (PE) foil with a light-permeability of 90% for an optimal light perception. After each drought period the rain-out shelters were removed and compensation irrigations with tap water were added by simulating a heavy rainfall event (Fig. S1) using a portable irrigation system. According to the naturally occurring water amounts during each drought treatment 102.5 mm water was added to the plots after D1 treatment and 187.5 mm after D2 treatment. To prevent water-run-off by simulated heavy rainfall events, the irrigation was divided into two applications on one day. To avoid lateral surface water flow plastic sheet pilings were placed around each plot down to a depth of 0.2–0.25 m.

Samples were taken at the last day of the drought (t0) and one, two and four weeks after the simulated heavy rainfall event (t1, t2, and t3). At each sampling date, three soil samples were collected from each of the five replicate plots per treatment with a corer of 3-cm diameter and pooled (5–15 cm depth; 0–5 cm were discarded because of a high root content). After each sample soil, the corer was sterilized with 70% Ethanol to prevent cross-contamination. All samples were immediately frozen at −80 °C until further processing for molecular analyses or stored at 4 °C for analyses of soil chemical parameters.

### Soil moisture

A continuous soil moisture measurement was performed every hour by using a frequency domain (FD)-sensors (ECH_2_O, Decagon devices, Pullman, USA) as described by Walter *et al*.^53^. Each logger was installed in each plot in undisturbed soil in a depth of −2 to −7 cm, respectively. The soil moisture data was calculated with means of each replicate per treatment (C, D1 and D2) and given in volume % [vol. %]. Additionally, soil moisture of sampled soils was determined by drying 2 g of fresh soil at 105 °C for 24 hours in an aluminum bowl and given per gram dry weight [g^−1^ dw].

### Soil chemical parameters

In order to determine the content of ammonium (NH_4_^+^-N), and nitrate (NO_3_^−^-N), soil samples were extracted by shaking 5 g fresh soil with 20 mL of 1 M KCl for 30 min on a rotary shaker. The soil suspension was filtered using 0.45 µm pore-size filters (Whatman International LTD, VWR, Germany), diluted 1:100 using sterile MilliQ water and stored at −20 °C until further analyses. The contents of ammonium (NH_4_^+^-N) and nitrate (NO_3_^−^-N) were determined by a continuous flow analysis with a photometric autoanalyzer (CFA-SAN Plus, SkalarAnalytik, Germany)^35^.

### DNA extraction

DNA was extracted from 0.5 g fresh soil using the FastDNA^®^ SPIN Kit for Soil (MP Biomedicals, Canada) and the Precellys24 Instrument (Bertin Technologies, France) according to the manufacturer’s protocol. After extraction, DNA quantity and quality were determined using the spectrophotometer Nanodrop (PeqLab, Germany) (73–214 ng µL^−1^ DNA). Subsequently, DNA was stored at −20 °C until further processing.

### Quantitative Real-Time PCR Assay

Quantitative Real-Time PCR (qPCR) for all marker genes used to describe ammonia oxidizers as well as denitrifiers was carried out on a 7300 Real-Time PCR System (Applied Biosystems, Germany) using SYBR green as fluorescent dye as previously described^56^. The PCR was performed in 96-well plates (Applied Biosystems, Germany): Details on marker genes and PCR conditions are described in Table 2. A dilution series of DNA extracts were tested in a pre-experiment to avoid the inhibition of PCR, resulting in an optimal dilution of 1:64 for all samples. The efficiencies (Eff) of the amplifications were calculated as described in Töwe *et al*.^56^ and resulted in the following values: *amoA* of ammonia-oxidizing bacteria (AOB) 93–95%, *amoA* of ammonia-oxidizing archaea (AOA) 91–98%, *nirS* 99–100%, *nirK* 94–99% and *nosZ* 84–85%. The specificity of the amplified products was checked by melting curves of the amplicons and agarose gels.

**Table 2:** Protocols for quantitative real-time PCR with thermal profiles, primers and standards used for the different functional genes.

Target gene	Thermal profile	No. of cycles	Primer	Source of standard
*amoA* AOA	94 °C–45 s/55 °C–45 s/72 °C–45 s	40	amo19F crenamoA16r48x^42,61^	Fosmid clone 54d9
*amoA* AOB	94 °C–60 s/58 °C–60 s/72 °C–60 s	40	amoA1F amoA2R^62^	*Nitrosomonas* sp.
*nirK*	95 °C–15 s/63 °C–30 s/72 °C–30 s	5^a^	nirK876 nirK5R^63,64^	*Azospirillum irakense*
	95 °C–15 s/58 °C–30 s/72 °C–30 s	40		
*nirS*	94 °C–45 s/57 °C–45 s/72 °C–4 s	40	cd3aF R3cd^65,66^	*Pseudomonas stutzeri*
*nosZ*	95 °C–15 s/65 °C–30 s/72 °C–30 s	5^a^	nosZ2F nosZ2R^67^	*Pseudomonas fluorescens*
	95 °C–15 s/60 °C–30 s/72 °C–30 s	40		

^a^Touchdown: −1 °C of the primer annealing temperature per cycle.

### Terminal restriction fragment length polymorphism

The diversity of the ammonia oxidizing archaea was assessed by using terminal restriction fragment length polymorphism (t-RFLP) of the *amoA* gene as described by Töwe *et al*.^57^. Same primers and PCR conditions were used for t-RFLP as described above for qPCR, but the forward primer was labeled with 5´-FAM (6-carboxyfluorescein) (Table 2). For the digestion of the PCR product the restriction enzyme *Mwo*I (New England BioLabs, Germany) was used as described by Bannert *et al*.^35^. Sequencing was performed on an ABI 3730 DNA Analyzer (Applied Biosystems, USA) and chromatograms were analyzed by using the GeneMapper 3.5 software package (Applied Biosystems, Germany) and T-REX software (http://trex.biohpc.org/) with a binning range of 1 basepare (bp). Data were normalized to percent of the total peak height of a sample. Fragments smaller than 50 bases and terminal restriction fragments (TRFs) contributing <0.5% to the total peak height were excluded^57^. Samples with peak height smaller than 3% were combined and marked as “others”. TRFs were assigned to the *amo*A_AOA Feifei-Liu reference database from FunGene^58^ using TRiFLe software^59^.

### Statistical analysis

Statistical analysis was performed using R 3.4.1^60^. Normal distribution was tested with the Kolmogorov-Smirnoff test; if needed, the data were log-transformed before further analyses. Because of repeated measurements, a linear mixed effect model was calculated (function lme in R-package nlme) and in case of a significant time effect pairwise comparisons were conducted by a Tukey test (function glht in R-package multcomp). To determine treatment-specific differences, we subsequently tested for effects of drought on soil chemical parameters and terminal restriction fragments by comparing treatment with respective controls over the study time by repeated-measures ANOVA. In order to describe treatment effects at single sampling points, we used a paired student’s t-test for independent samples. Changes over time between C and D1 or C and D2 and changes due to the treatment were analyzed with a univariate analysis of variances.

## Supplementary information

Supplementary Figure S1

## Data Availability

The datasets generated during and/or analyzed during the current study are available from the corresponding author on reasonable request.
