# Supplementary material for: Influence of rewetting on microbial communities involved in nitrification and denitrification in a grassland soil after a prolonged drought period
Source: Sci Rep. 2019 Feb 19;9:2280. doi: 10.1038/s41598-018-38147-5 (PMC6381133; doi:10.1038/s41598-018-38147-5)

Influence of rewetting on microbial communities involved in nitrification and denitrification in a grassland soil after a prolonged drought period

Verena Hammerl[1,2], Eva-Maria Kastl[1], Michael Schloter[1], Susanne Kublik[1], Holger Schmidt[3], Gerhard Welzl[1], Anke Jentsch[4], Carl Beierkuhnlein[5], Silvia Gschwendtner[1*]

[1] Research Unit Comparative Microbiome Analysis - Helmholtz Zentrum München, Ingolstädter Landstr. 1, 85764 Neuherberg, Germany

[2] Chair for Soil Ecology - Technische Universität München, Ingolstädter Landstr. 1, 85764 Neuherberg, Germany

[3] Institute of Natural Sciences - Universität Koblenz Landau, Campus Koblenz, Universitätsstraße 1, 56070 Koblenz, Germany

[4] Disturbance Ecology - University of Bayreuth, Universitätsstr. 30, 95447 Bayreuth, Germany

[5] Chair of Biogeography - University of Bayreuth, Universitätsstr. 30, 95447 Bayreuth, Germany

* Corresponding author: silvia.gschwendtner@helmholtz-muenchen.de

Supplemental Material

Figure S1.

Soil moisture [vol. %] and natural precipitation per day [mm]. Soil moisture data are shown with lines, for C (black grey line), D1 (light grey line) and D2 (dark grey line). Natural precipitation data are shown with bars. Light grey line indicates the duration of D1 treatment and dark grey line the duration of D2 treatment. Compensation irrigations during the experimental time are marked with black arrows. Sampling time points are marked with triangles (light grey triangles for D1 and dark grey triangles for D2).

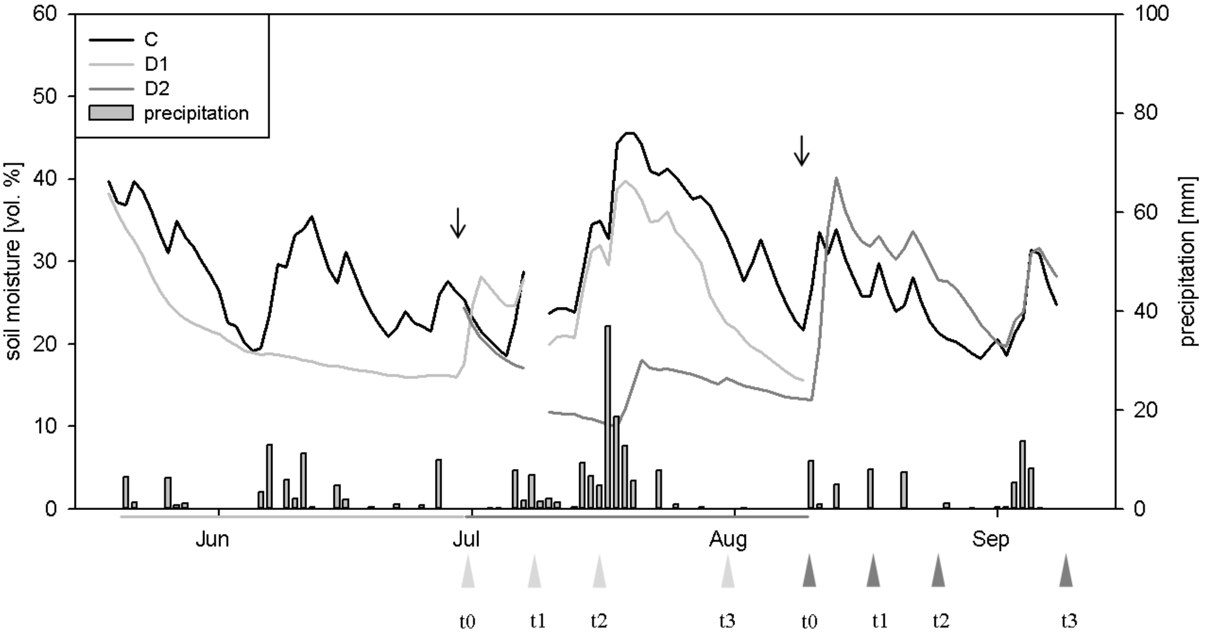

Supplement: Supplementary file 1 — Supplementary Figure S1 [file 41598_2018_38147_MOESM1_ESM.pdf]
